# Perikarditis im Rahmen rheumatologischer Erkrankungen – Was der Rheumatologe wissen sollte

**DOI:** 10.1007/s00393-020-00925-w

**Published:** 2020-11-20

**Authors:** M. Krusche, U. Schneider, N. Ruffer

**Affiliations:** 1 Medizinische Klinik mit Schwerpunkt Rheumatologie und Klinische Immunologie, Charité Universitätsmedizin Berlin, Charitéplatz 1, 10117 Berlin, Deutschland; 2 Klinik für Rheumatologie und Immunologie, Klinikum Bad Bramstedt, Bad Bramstedt, Deutschland

**Keywords:** Perikarditis, Idiopathische rekurrierende Perikarditis, Autoinflammation, Antiinflammatorische Therapie, Colchicin, Pericarditis, Idiopathic recurrent pericarditis, Autoinflammation, Anti-inflammatory therapy, Colchicin

## Abstract

Als Perikarditis wird eine Entzündung des Herzbeutels bezeichnet, die mit einem Perikarderguss oder einer entzündlichen Reaktion des Myokards (Perimyokarditis) einhergehen kann. Die Perikarditis kann im Rahmen von entzündlich rheumatischen Systemerkrankungen oder als eigenständige Erkrankung vorkommen. Rezidivierende Perikarditisepisoden ohne fassbare Ursache werden als idiopathische rekurrierende Perikarditis (IRP) bezeichnet, welche wesentliche Gemeinsamkeiten mit autoinflammatorischen Erkrankungen aufweist. Der Artikel gibt einen Überblick über die Häufigkeit des Auftretens einer Perikarditis bei rheumatologischen Erkrankungen. Weiterhin werden Klinik und Pathophysiologie der IRP diskutiert. Abschließend wird die Therapie der akuten und idiopathischen Perikarditis erläutert.

## Abstract (en)

Pericarditis is the term for inflammatory involvement of the pericardium, which can be associated with pericardial effusion and myocardial involvement (perimyocarditis). Pericarditis can be present in the context of systemic inflammatory rheumatic diseases but can also constitute a distinct disease entity. Idiopathic recurrent pericarditis (IRP) describes relapsing conditions of pericarditis with an unknown cause, which show essential common features with autoinflammatory diseases. This article gives an overview of the frequency of pericarditis in systemic rheumatic diseases. Moreover, the clinical manifestations and pathophysiology of IRP are discussed. Finally, the therapeutic algorithms for acute and idiopathic pericarditis are explained.

Als Perikarditis wird eine Entzündung des Herzbeutels bezeichnet, die mit einem Perikarderguss oder einer entzündlichen Reaktion des Myokards (Perimyokarditis) einhergehen kann. Entzündlich rheumatische Systemerkrankungen sind häufig mit einer Perikarditis assoziiert. Eine Perikarditis kann jedoch auch als eigenständige Erkrankung auftreten. Für den Rheumatologen sind die differenzialdiagnostische Einordnung dieses Symptomes bzw. Krankheitsbildes sowie die adäquate Therapie in der Praxis von großer Bedeutung [1].

## Akute Perikarditis

Die Diagnose einer akuten Perikarditis kann in der Zusammenschau klinischer, laborchemischer, elektrokardiographischer und bildmorphologischer (meist sonographisch) Befunde gestellt werden. Gemäß der aktuellen European Society of Cardiology(ESC)- und der Deutschen Gesellschaft für Kardiologie – Herz- und Kreislaufforschung e. V.(DGK)-Leitlinie müssen dabei mindestens 2 der 4 folgenden Kriterien erfüllt sein:

- perikarditische Thoraxschmerzen,
- Perikardreiben,
- EKG-Veränderungen: neue ST-Hebungen in vielen Ableitungen oder PR-Senkungen,
- neuer oder zunehmender Perikarderguss.

Zusätzliche unterstützende Befunde sind:

- laborchemische Hinweise für eine systemische Entzündung (C-reaktives Protein, CRP; Blutsenkungsgeschwindigkeit; Leukozytose),
- Nachweis eines Perikardergusses durch bildgebende Verfahren (Echokardiographie, Computertomographie, kardiale Magnetresonanztomographie).

Wesentliche Indikatoren für einen ungünstigen Verlauf (im Sinne einer längeren Hospitalisierungsdauer und/oder Komplikationen) sind Fieber (>38 °C), ein subakuter Krankheitsbeginn, ein großer Perikarderguss, eine Herzbeuteltamponade sowie ein schlechtes Therapieansprechen auf nichtsteroidale Antirheumatika (NSAR) [2].

Eine Perikarditis kann im Rahmen verschiedener rheumatologischer Erkrankungen, (z. B. systemischer Lupus erythematodes, systemische Sklerose, rheumatoide Arthritis oder im Rahmen von autoinflammatorischen Syndromen; Tab. 1) auftreten. Die Beteiligung des Perikards kann bei diesen Erkrankungen symptomatisch (Perikarditis bzw. symptomatischer Perikarderguss) oder asymptomatisch (meist kleiner Perikarderguss) verlaufen. Im Allgemeinen spiegelt die Perikarditis den Aktivitätsgrad der Grunderkrankung wider, tritt jedoch auch selten isoliert als Erstsymptom auf.

**Table 1:** 

Erkrankung	Auftretenshäufigkeit	Quelle
„Adult onset Still’s disease“	3–37 %	[45]
Morbus Behçet	<5 %	[46]
Systemischer Lupus erythematodes	Ca. 25 % symptomatisch >50 % asymptomatisch	[47]
Systemische Sklerose	Perikarditis 1,9–9 % Perikarderguss 15–72 %	[48]
Eosinophile Granulomatose mit Polyangiitis	Perikarderguss ca. 6–7 %	[49]
Granulomatose mit Polyangiitis	Ca. 5 %	[50, 51]
Mikroskopische Polyangiitis	6 %	[52]
Panarteriitis nodosa	Ca. 5–6 %	[53, 54]
Kawasaki-Syndrom	3–16 %	[55, 56]
Kardiale Sarkoidose	<5 %	[57]
Familiäres Mittelmeerfieber	2–30 %	[58, 59]
TNF-Rezeptor-assoziiertes periodisches Syndrom	7 %	[11]

Neben autoimmunen Krankheitsbildern sind v. a. Infektionen (insbesondere viraler Genese), aber auch das Postperikardektomiesyndrom, metabolische Störungen, Neoplasien, Traumata oder medikamentös toxische Ursachen als Perikarditisäuslöser möglich (Tab. 2).

**Table 2:** 

Infektion	
Viral (häufig)	Enteroviren (Coxsackie, Echoviren), Herpesviren (EBV, CMV, HHV-6), Adenoviren, Parvovirus B19
Bakteriell	*Mycobacterium tuberculosis* (häufig), *Coxiella burnetii*, *Borrelia burgdorferi* Selten: *Pneumococcus *spp., *Meningococcus *spp., *Gonococcus *spp., *Streptococcus *spp., *Staphylococcus *spp., *Haemophilus* spp., *Chlamydia *spp., *Mycoplasma *spp., *Legionella *spp., *Leptospira *spp., *Listeria *spp., *Providencia stuartii*
Pilze (sehr selten)	*Histoplasma *spp. (eher bei Immunkompetenten), *Aspergillus *spp., *Blastomyces *spp., *Candida *spp. (eher bei Immunkomprimierten)
Parasiten (sehr selten)	*Echinococcus *spp., *Toxoplasma *spp.
Nichtinfektiöse Ursachen	
Autoimmun	*Vgl. *Tab. 1
Neoplasien	Primärtumore (selten, a. e. perikardiale Mesotheliome) Sekundär Metastasen (häufig, v. a. Lungen- und Mammakarzinom, Lymphom)
Metabolisch	Urämie, Myxödem, Anorexia nervosa
Trauma/iatrogen	*Früher Beginn (selten)*
	Direkte Verletzung (penetrierende Thoraxverletzung, Ösophagusperforation)
	Indirekte Verletzung (nicht penetrierende Thoraxverletzung, Strahlenschäden)
	*Verzögerter Beginn (häufig)*
	Dressler-Syndrom, Postperikardektomiesyndrom
Medikamententoxisch (selten)	Lupus-like-Syndrome (Procainamid, Hydralazin, Methyldopa, Isoniazid, Phenytoin); Chemotherapeutika (oft mit Kardiomyopathie assoziiert): Doxorubicin, Daunorubicin, Cytosin-Arabinoside, Cyclophosphamid, 5‑Fluorouracil; Penicilline als Hypersensitivitätsperikarditis mit Eosinophilie; Amiodaron, Methysergid, Mesalazin, Clozapin, Minoxidil, Dantrolen, Practolol, Phenylbutazon, Thiazide, Streptomycin, Thiouracil, Streptokinase, p‑Aminosalicylicsäure, Cyclosporin, Bromocriptin, GM-CSF, TNF-Blocker
Andere Ursachen (selten)	Amyloidose, Aortendissektion

Differenzialdiagnostisch sollte bei Patienten aus den Tuberkuloseendemiegebieten an das Vorliegen einer Mykobakteriose gedacht werden (Ursache ca. 75 % aller Perikarderkrankungen in Ländern der Dritten Welt) [3].

Komplizierend kann in ca. 15 % der Fälle mit akuter Perikarditis auch das Myokard im Sinne einer Perimyokarditis beteiligt sein. Insbesondere bei jungen Patienten, männlichem Geschlecht, Arrhythmien oder ST-Streckenveränderungen sowie Fieber sollte an das zusätzliche Auftreten einer Myokarditis gedacht werden. Durch die laborchemische Bestimmung von Troponin kann zwischen einer Myokarditis und einer isoliert vorliegenden Perikarditis diskriminiert werden, da dieses bei Letzterer typischerweise nicht erhöht ist.

Insgesamt hat das Erkrankungsbild der akuten Perikarditis jedoch eine gute Prognose und heilt bei den meisten Patienten ohne kardiale Folgeschäden vollständig aus [4]. Akutschäden wie eine Herzbeuteltamponade oder auch Folgeschäden wie eine konstriktive Perikarditis sind sehr selten (<1%) [5]. Allerdings steigt die Rezidivrate nach der ersten Episode um bis zu 50 % (von 18,3 % auf 38,2 %), insbesondere bei Patienten, die mit Steroiden vorbehandelt wurden [6]. Ein unzureichendes Ansprechen auf NSAR (nach 1‑wöchiger Therapie) und eine persistierende CRP-Erhöhung sind ebenfalls mit einer erhöhten Rezidivrate verbunden [6].

## Idiopathisch rekurrierende Perikarditis

Von der akuten Perikarditis ist das Krankheitsbild der rekurrierenden Perikarditis (RP) abzugrenzen. Die RP ist über den Nachweis eines Perikarditisrezidivs nach Auftreten einer akuten Perikarditis und zwischenzeitlich symptomfreiem Intervall von 4 bis 6 Wochen definiert. Eine RP kann in bis zu 30 % der Fälle infolge einer akuten Perikarditis auftreten [7].

### Ätiologie

In ca. 80 % der Fälle einer rekurrierenden Perikarditis lässt sich keine Krankheitsursache ermitteln, weshalb der Begriff idiopathische rekurrierende Perikarditis (IRP) verwendet wird. Bei einem gewissen Anteil der Erkrankungen können virale Infektionen [3] als Krankheitstrigger ausgemacht werden (Tab. 2). Eine umfassende virale Erregerdiagnostik ist aber in der Praxis selten sinnvoll, da sich hieraus in der Regel keine therapeutische Konsequenz ergibt [8].

In der laborchemischen Autoimmundiagnostik lassen sich häufig unspezifische ANA-Titer nachweisen [9]. Da eine Perikarditis, wie bereits erwähnt, im Rahmen von rheumatologischen Systemerkrankungen auftreten kann, sollten insbesondere in diesen Fällen eine entsprechende Systemanamnese sowie ggf. eine ergänzende immunserologische Testung zur differenzialdiagnostischen Abgrenzung erfolgen (Tab. 3).

**Table 3:** 

Klinische Merkmale	Virale Infektion	Idiopathische Perikarditis	Autoinflammatorisches Syndrom	Kollagenose
Geschlecht (w/m)	1:1	1:1	1:1	8:2
Krankheitsbeginn (Alter)	Jedes	Jedes	<20 Jahre	Jedes
Arthralgien/Myalgien	+	−	±	++
Fieber	+	+	+	+
Hautausschlag	+	−	+	+
Pleuritis	±	++	+	+
Exsudat	+	++	±	+
Dauer der Krankheitsepisode >4 Wochen	–	+	±	+
Saisonales Auftreten	Späte Winter	Akut: Januar bis März (wiederkehrend kein Pattern)	−	Sommer
ANA >1:320	−	−	−	++
Ansprechen auf Glukokortikoide	−	+	±	++
Ansprechen auf Colchicin	−	+	FMF + CAPS/TRAPS −	−
Ansprechen auf Anti-IL‑1	−	++	++	−

− abwesend, ± selten, + häufig, ++ sehr häufig

Insbesondere autoinflammatorischen Syndrome wie das familiäre Mittelmeerfieber (FMF) und das TNF-Rezeptor-assoziierte periodische Syndrom (TRAPS) können mit einer Perikarditis einhergehen. So konnte in Registerdaten von 346 pädiatrischen FMF-Patienten eine Perikarditis in 18 % der Fälle nachgewiesen werden. Interessanterweise berichteten in der Studie sogar 56 % der Patienten über Brustschmerzen [10]. Die Häufigkeit des Auftretens einer Perikarditis bei TRAPS wird auf ca. 7 % geschätzt [11].

Einige Autoren empfehlen eine TRAPS-Testung bei positiver Familienanamnese für eine IRP, da familiäre Häufungen bei unvollständigen TRAPS-Phänotypen mit Mutationen niedriger Penetranz beschrieben wurden [12]. So konnte in einer italienischen Studie an 131 kaukasischen IRP-Patienten bei 6,1 % eine Mutation des *TNFRSF1A*-Gens nachgewiesen werden [13]. Bei diesen Patienten bestand überwiegend ein schlechtes therapeutisches Ansprechen auf Colchicin, und es zeigten sich vermehrte Krankheitsrezidive, die den Einsatz weiterer Immunsuppressiva erforderten [13]. Cantarini et al. [14] konnten für die TRAPS-Mutationsvarianten mit niedriger Penetranz (R92Q, P46L, D12E, V95 M und R104Q) eine erhöhte Perikarditisprävalenz nachweisen. Bei diesen Patienten war der Krankheitsbeginn jedoch später, und es gab weniger schwerwiegende entzündliche Schübe.

Brucato und Brambila [15] konnten weiterhin zeigen, dass Angehörige von IRP-Patienten ein erhöhtes Risiko für die Entwicklung einer Perikarditis aufweisen.

### Pathophysiologie der IRP

Die Pathophysiologie der IRP ist bisher nicht vollständig aufgeklärt. Autoimmunologische und autoinflammatorische Prozesse scheinen jedoch eine wesentliche Rolle zu spielen. Imazio [16] vermutet ein Zusammenspiel von Umweltfaktoren mit angeborener und adaptiver Immunität auf dem Boden einer entsprechenden genetischen Prädisposition.

#### Autoimmunität

Möglicherweise kommt der Antigenpräsentation bei T‑Zellen eine Bedeutung bei der Entstehung der IRP zu. Hinweise hierfür fanden sich in einer Kohorte von 55 griechischen Patienten mit IRP [17]. Hier zeigten sich eine erhöhte Häufigkeit der HLA-Allele HLA-A*02, HLA-Cw07 und HLA-DQB1*0202 sowie eine verminderte Häufigkeit von HLA DQB1*0302.

Ein weiterer Hinweis für das Vorhandensein einer autoimmunologischen Komponente ist das häufige Vorkommen von ANA, welche in einer Studie bei 43,3 % der IRP-Patienten nachgewiesen werden konnten [18]. Weiterhin konnte in einer Studie bei 67,5 % der IRP-Patienten das Auftreten von „anti-heart“ (AHA) und/oder „anti-intercalated disk autoantibodies“ (AIDA) gezeigt werden [19]. Inwieweit diese Antikörper eine klinische Bedeutung haben oder nur ein Epiphänomen sind, ist jedoch weiterhin unklar [20].

#### Autoinflammation

Durch ein genaueres Verständnis von autoinflammatorischen Erkrankungen und die verbesserten humangenetischen Testmethoden konnten in den letzten Jahren viele Fortschritte in diesem Forschungsfeld erzielt werden. In ihrem klinischen Erscheinungsbild zeigt die IRP zumeist wesentliche Gemeinsamkeiten mit anderen autoinflammatorischen Erkrankungen wie ein unprovoziertes Auftreten von Krankheitsschüben in Abwesenheit von antigenspezifischen T‑Zellen oder (hochtitrigen) Autoantikörpern [21].

Die hierfür entscheidende Struktureinheit ist das Inflammasom, eine makromolekulare intrazelluläre Plattform, welche als ein angeborener Immunitätssensor fungiert [22]. Unter allen identifizierten Inflammasomen ist das NALP3-Inflammasom am besten charakterisiert. NALP3 ist eine makromolekulare Struktur, bestehend aus dem NOD-like-Rezeptor(NLR)-Protein, dem Adapter ASC und Caspase 1. Es wird durch ein breites Spektrum von pathogen- (PAMPS) oder „danger“-assoziierten molekularen Patterns (DAMPS) aktiviert und induziert über die Spaltung von Pro-Interleukin 1β zu aktivem Interleukin 1β eine inflammatorische Kaskade [23]. Interessanterweise sind einige kardiotrope Viren wie Adenoviren, Influenza A, Herpesviren und das Cytomegalovirus in der Lage, NALP3 und andere Inflammasome zu aktivieren [24–26].

Bezüglich der proinflammatorischen Zytokine, die an der Pathogenese der Perikarditis beteiligt sind, gibt es nur wenige Daten. Eine kleine Studie zeigte, dass bei Patienten mit autoreaktiver Perikarditis (definiert als Ausschluss einer Neoplasie, Infektion oder systemischen Ursachen) erhöhte Interleukin-6-Spiegel in der Perikardflüssigkeit nachweisbar waren [27]. In anderen Arbeiten konnten neben erhöhten IL-6-Spiegeln auch erhöhte IL-8- und Interferon(IFN)-γ-Spiegel im Perikarderguss nachgewiesen werden [17].

## Therapie der akuten (nichtinfektiösen) und idiopathischen rekurrierenden Perikarditis

**Figure 1:**
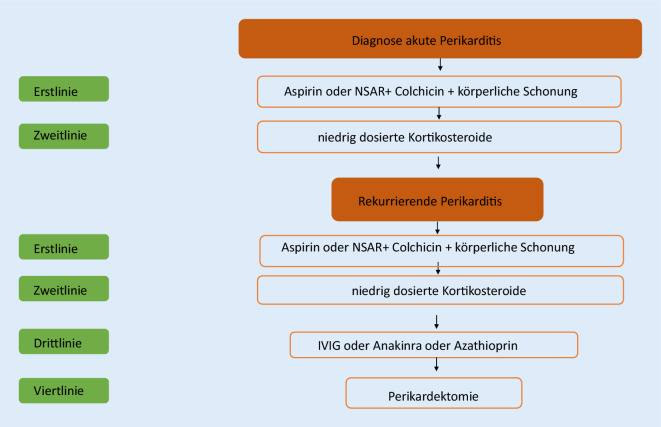

Eine Übersicht zur Therapie der beiden Perikarditisformen zeigt Abb. 1.

### Erstlinientherapie

#### NSAR

Zur Erstlinientherapie der akuten und rezidivierenden Perikarditis werden NSAR eingesetzt (Tab. 4). Basierend auf einer älteren randomisierten Studie mit 149 Patienten mit Postperikardiotomiesyndrom werden Ibuprofen oder Indometacin empfohlen. Beide Medikamente waren in einer Studie im Vergleich zu Placebo innerhalb von 48 h wirksam und linderten die Krankheitssymptome [28].

**Table 4:** 

Medikament	Dosierung	Dauer	Tapering
Aspirin	500–1000 mg alle 6–8 h	Wochen bis Monate	Reduktion der Dosis um 250–500 mg alle 1 bis 2 Wochen
Ibuprofen	600 mg alle 8 h	Wochen bis Monate	Reduktion der Dosis um 200–400 mg alle 1 bis 2 Wochen
Indometacin	25–50 mg alle 8 h	Wochen bis Monate	Reduktion der Dosis für um 25 mg alle 1 bis 2 Wochen
Colchicin	0,5 mg 2‑mal täglich oder 0,5 mg 1‑mal täglich für Patienten <70 kg oder mit Intoleranz für höhere Dosen	Mindestens 6 Monate	Nicht nötig Alternativ 0,5 mg jeden zweiten Tag oder 0,5 mg 1‑malig (>70 kg) in den letzten Wochen

Es wird empfohlen NSARs bis zum Abklingen der Symptome und Normalisierung der Entzündungsmarker einzusetzen. Bei hoher Dosierung ist eine zusätzliche Gastroprotektion mit einem Protonenpumpenhemmer ratsam.

#### Colchicin

Der antiinflammatorische Wirkmechanismus von Colchicin ist zentral für die Überlegung, dass die IRP Merkmale einer autoinflammatorischen Erkrankung trägt: Colchicin beeinflusst direkt und indirekt die Regulation von autoinflammatorischen Zytokinsignaturen. Es hemmt die Polymerisation von Mikrotubuli und verhindert somit die Freisetzung chemotaktischer Faktoren aus neutrophilen Granulozyten. Dosisabhängig hemmt Colchicin die Expression von E‑Selektin auf Endothelzellen sowie die Adhäsion von Neutrophilen. Außerdem fördert es den Abbau von L‑Selektin aus Neutrophilen, wodurch deren weitere Rekrutierung gebremst wird. Colchicin verhindert weiterhin die Aktivierung der P2X2- und P2X7-Poren, welche wiederum ATP-induziert NALP‑3 aktivieren. Darüber hinaus hemmt es direkt die Freisetzung von TNF‑α, NO und reaktiven Sauerstoffspezies [29].

In klinischen Studien konnte gezeigt werden, dass Colchicin nachweislich die Behandlungsdauer einer IRP verkürzt, die Remissionsdauer verlängert und das Rezidivrisiko um ca. 50 % senkt [30–32]. Die Arbeiten von Imazio et al. und die Daten einer großen Metaanalyse [33] waren die Grundlage dafür, dass Colchicin in den European Society of Cardiology(ESC)-Leitlinien als Erstlinientherapie der IRP in einer Dosierung von 0,5 mg 2‑mal täglich für mindestens 6 Monate empfohlen wird [2].

### Zweitlinientherapie

#### Kortikosteroide

Kortikosteroide können bei akuter und rezidivierender Perikarditis eingesetzt werden. Allerdings sind insbesondere hohe Dosen (≥1 mg/kgKG pro Tag) mit einem erhöhten Rezidivrisiko assoziiert [34] und sollten daher vermieden werden. Für Patienten, bei denen NSAR und/oder Colchicin nicht ausreichend wirksam oder kontraindiziert sind, können niedrigere Prednisolon-Dosen (0,2–0,5 mg/kg/Tag) eingesetzt werden [35]. Die Prednisolon-Reduktion sollte in kleinen Schritten alle 2 bis 4 Wochen erfolgen (Tab. 5).

**Table 5:** 

Startdosis: 0,25–0,5 mg/kg/Tag	Tapering^a^
>50 mg	Um 10 mg alle 1 bis 2 Wochen
50–25 mg	5–10 mg alle 1 bis 2 Wochen
25–15 mg	2,5 mg alle 2 bis 4 Wochen
<15 mg	1,25–2,5 mg alle 2 bis 6 Wochen

^a^Prednisolon-Reduktion nur, wenn der Patient asymptomatisch und das CRP normalisiert ist

### Drittlinientherapie

Bei einem geringen Anteil der Patienten, die Kortikosteroide benötigen, ist eine adäquate Steroidreduktion nicht möglich: Zur steroidsparenden Therapie kann *Azathioprin* eingesetzt werden. Die Evidenzlage hierfür ist jedoch überschaubar. In einer retrospektiven Studie mit 46 Patienten, in der Azathioprin in einer Dosis von 1,5–2,5 mg/kg/Tag über 1 Jahr gegeben wurde, konnte eine stabile Remission erzielt werden, und bei über 50 % der Patienten war ein komplettes Absetzen möglich [36].

Ebenfalls können bei refraktärer IRP *intravenöse Immunglobuline (IVIG)* eingesetzt werden. So konnten Imazio et al. bei 30 Patienten mit IRP ein gutes Ansprechen auf IVIG zeigen [37]. Die IVIG wurden in der Studie über 3 bis 5 Tage mit einer Dosis von 400–500 mg/kgKG pro Tag infundiert.

#### Interleukin-1-Antagonisten

Die beste Studienlage für die Tertiärtherapie der IRP liegt für den Interleukin-1(IL-1)-Rezeptorantagonisten *Anakinra* vor. Anakinra führte in Studien zu einer raschen Besserung der Symptome innerhalb von Tagen (im Gegensatz zu Wochen bei Anwendung von Azathioprin). Weiterhin zeigen sich unter der Therapie eine rasche Normalisierung der serologischen Entzündungszeichen und ein bildmorphologisches Ansprechen (Echokardiographie und Kardio-MRT) [38].

In einer doppelblinden, placebokontrollierten, randomisierten Studie, die an 21 steroidabhängigen, Colchicin-resistenten Patienten durchgeführt wurde, reduzierte Anakinra das Risiko eines Rezidivs über einen Median von 14 Monaten und ermöglichte bei allen Patienten das Absetzen der Steroide [39].

In der Registerarbeit des International Registry of Anakinra for Pericarditis (IRAP) konnte bei 224 IRP-Patienten, die Colchicin-resistent und Kortikosteroid-abhängig waren, gezeigt werden, dass der Einsatz von Anakinra u. a. eine Verringerung der Rezidivrate um 83 % und eine signifikante Kortikosteroidreduktion ermöglichte [40].

Erste Daten gibt es ebenfalls für weitere IL-1-Antagonisten: In einer kleinen Fallserie berichteten Kougkas et al. [41] über den erfolgreichen Einsatz von Canakinumab zur Behandlung von 3 IRP-Fällen. Für den IL-1-Antagonisten Rilonacept läuft aktuell eine randomisierte placebokontrollierte Phase-3-Studie. Erste vorläufig veröffentlichte Daten legen auch hier ein klinisches (Schmerzreduktion) und serologisches (CRP-Reduktion) Ansprechen nahe [42]. Die gute Wirksamkeit von IL-1-Antagonisten wird ebenfalls als Indiz für den autoinflammatorischen Charakter der IRP gewertet. Bezüglich der optimalen Therapiedauer mit IL-1-Antagonisten gibt es noch keine Empfehlungen.

### Viertlinientherapie

#### Perikardektomie

Bei unzureichender pharmakotherapeutischer Therapie kann eine Perikardektomie in Erwägung gezogen werden. In einer Studie konnten die positiven Auswirkungen einer subtotalen chirurgischen Perikardektomie für Patienten mit konstriktiver Perikarditis gezeigt werden [43]. Gillaspie et al. [44] konnten zeigen, dass eine radikale Perikardektomie bei IRP-Patienten häufiger zu einem Langzeitüberleben führte als eine partielle Perikardektomie mit zusätzlicher medikamentöser Therapie. In der untersuchten Kohorte betrug die Gesamtüberlebenszeit nach Perikardektomie 80 % nach 5 Jahren und 60 % nach 10 Jahren.

## Fallbeispiel

**Figure 2:**
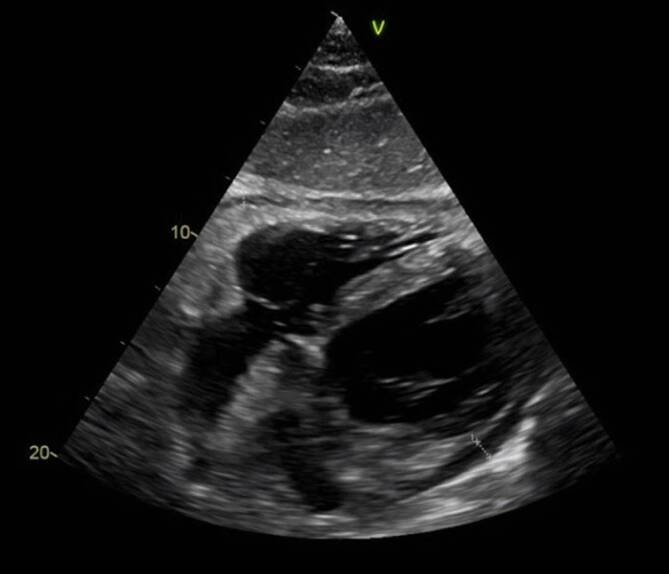

**Figure 3:**
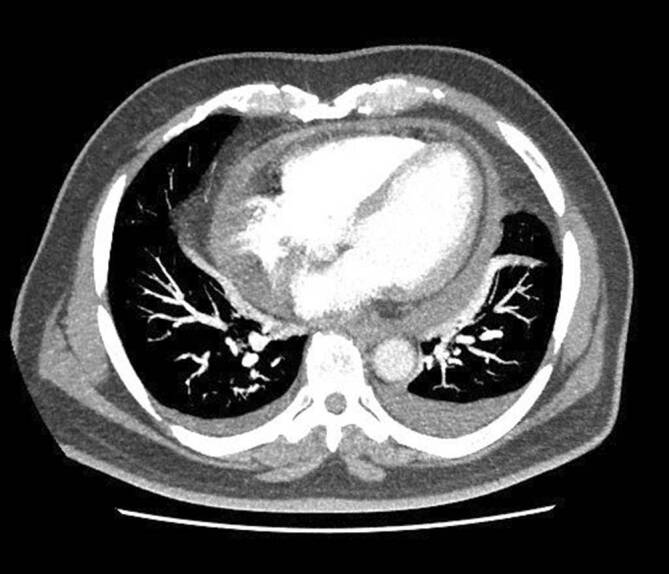

**Figure 4:**
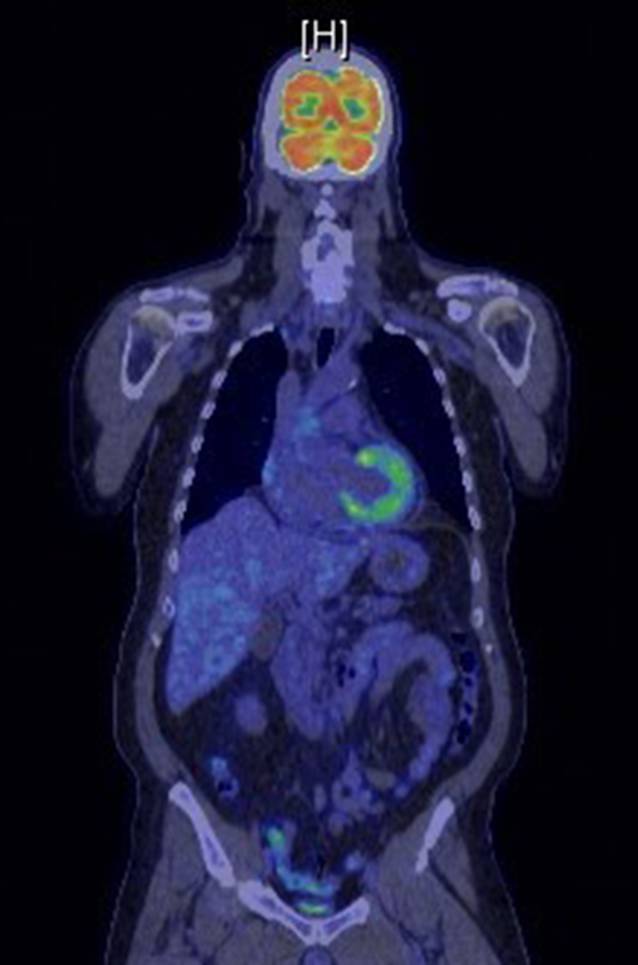

Wir schildern den Fall eines 67-jährigen Patienten mit idiopathischer rekurrierender Perikarditis. Der Patient beklagte Thoraxschmerzen mit Fieber (bis 38,6 °C im Schub). Bildmorphologisch sah man einen Perikarderguss von bis zu 1,6 cm (Abb. 2). Der Patient hatte insgesamt 3 IRP-Episoden in 8 Monaten (initial sogar mit beidseitiger Pleuritis; Abb. 3). Laborchemisch zeigten sich im Schub eine CRP-Erhöhung von bis zu 302,9 mg/l (normal <5 mg/l) und ein erhöhtes Serumamyloid A von 1540 mg/l (normal <6,4 mg/l). Die Autoimmunserologie (RF, ACPA, ANA, ENA, ANCA) war unauffällig. Mittels PET-CT konnte ein weiterer Entzündungsfokus oder solitäres Malignom ausgeschlossen werden (Abb. 4).

Bei unzureichender Wirksamkeit von Ibuprofen, Colchicin und Prednisolon erfolgte eine Umstellung auf den IL-1-Rezeptorantagonist Anakinra 100 mg s.c. täglich. Hierunter zeigte sich ein promptes klinisches, laborchemisches und sonographisches Therapieansprechen. Bei anhaltender Symptomfreiheit wurde Anakinra nach 6 Monaten abgesetzt. Innerhalb von Tagen nach dem Absetzen bekam der Patient ein erneutes Rezidiv, das ebenfalls erfolgreich mit Anakinra behandelt werden konnte.

## Fazit für die Praxis

- Eine Perikarditis kann im Rahmen von rheumatologischen Systemerkrankungen auftreten, daher sollten eine entsprechende Systemanamnese sowie ggf. ergänzende immunserologische Testung erfolgen.
- Die idiopathische rekurrierende Perikarditis (IRP) wird von vielen Autoren als eigenständige autoinflammatorische Erkrankung gewertet. Eine autoimmunologische Pathogenese ist jedoch in einigen Fällen ebenfalls möglich.
- Die IRP sollte primär antiinflammatorisch mit NSAR und Colchicin behandelt werden.
- Erst bei primärem Therapieversagen (oder Kontraindikationen) sollten Kortikosteroide zur Behandlung der IRP zum Einsatz kommen.
- Kortikosteroide sollten zur Therapie der IRP nur in moderaten Dosen eingesetzt werden (0,2–0,5 mg/kgKG pro Tag), da ansonsten ein erhöhtes Rezidivrisiko besteht.
